# Memoirs of the Royal Medical Society at Paris

**Published:** 1782

**Authors:** 


### II. *Memoirs of the Royal Medical Society at Paris.*
(Continued from page 293.)

INQUIRIES *concerning St. Anthony's Fire. By* Meſſieurs de Juſſieu, Paulet, Saillant, and Abbé Teſſier. This hiſtorical inquiry is intended to prove, among other things, that the *peſtis inguinalis*, *mal des ardens* of Guy de Chauliac and other French writers, is different from St. Anthony's fire (*feu Saint Antoine*), of which there appears to be no clear, diſtinct account in any of the old writers, and which ſeems to be of two ſpecies that have led to the diſtinctions of *gangræna ſicca* and *humida*. The former is ſaid to be generally the effect of ſmutty rye uſed as food. The methods of treatment are given from the beſt writers.——15. *Inquiries relative to an Epidemic Convulſion, attributed by ſome writers to ſmutty rye, and confounded with the dry gangrene. By* M. Saillant. The dry gangrene is characterized by the mortification of one or all of the extremities. The veſſels become obliterated, the fleſh black and hard, and the gangrene penetrates to the bones. But in the epidemic convulſion there is no gangrene. Violent Convulſions conſtitute the eſſential character of the diſeaſe. Both, however, have been attributed to the ſame

 cauſe, caufe, viz. fmutty rye. The firft mention of the epidemic convulfion was in 1597, when it appeared in Weftphalia. The medical faculty of Marbourg publifhed a treatife on it, in which they confidered it as the effect of bad diet. In 1661 it appeared in England, and is fpoken of by Willis *(de Morb. Convulf. cap.* 8.) who afcribes it to the conftitution of the air, and fuppofes its proximate caufe to be an impoverifhment of the nervous fluid. It has fince prevailed at different periods in Saxony, Germany, Alface, and other northern parts of Europe; and was obferved in Sweden in 1746 and 1747. It was there obferved that much *raphaniftrum* or *baftard radifh* grew among the barley in thofe years; and Linnæus having fed fome birds with it, and finding that they died convulfed, gave the difeafe the name of *raphania*. Several writers have fuppofed that the dry gangrene and the convulfions are only different fymptoms of the fame difeafe, which have appeared together or feparate according to time and other circumftances. The author of thefe inquiries queries whether the fame caufe which in cold climates produces convulfions, may not in warmer ones excite gangrene? He promifes to continue his inquiries on this fubject, by feeding different animals with with *raphaniſtrum*, and to inform us of the reſult.

——16. *An account of the Remedies moſt neceſſary for Sheep. By* M. Daubenton. Sheep, the author obſerves, reſiſt all the variations of the French climate except great heat, and hence they are ſubject to what is called *maladie de la chaleur*. In caſes of this ſort, the animal opens his mouth to get air, foams, bleeds at the noſe, breathes ſhort and quick, the globe of the eye becomes red, the animal hangs down his head, totters, and ſoon falls dead. If the body is examined after death, all the blood veſſels, eſpecially thoſe of the head, are found diſtended. The remedy for this diſeaſe is bleeding. Another diſeaſe to which ſheep are particularly liable, is the fly. The moſt favourable circumſtances of paſturage, care, &c. are not ſufficient, we are told, to exempt them from it. In general it requires only a topical application, and the beſt for this purpoſe is a mixture of ſuet and oil of turpentine. M. Daubenton recommends the bleeding ſheep in the lower part of the cheek, cloſe to the root of the fourth grinder. This ſpot may be aſcertained by a tubercle on the external ſurface of the upper jaw, which may be eaſily felt by the finger on the ſurface of the ſkin. The angular vein paſſes immediately under this tubercle.

tubercle. An engraving is added on this ſubject. ——17. *Memoir relative to the contagious diſorder among the Horned Cattle in Holland.* *By* M. Camper. It appears from this paper, that of the animals that have had this diſeaſe in the natural way two thirds have periſhed, whereas by inoculating calves the loſs has not exceeded two or three in a hundred. They are inoculated before they are four months old, and the ſymptoms are ſaid to be very ſlight. Our author adviſes a repetition of the operation, that we may be ſure the diſeaſe has taken place, becauſe, for want of this precaution, ſome have had it and died after having been thought ſecure. He has inoculated different animals with the matter of the ſmall-pox, but without any effect.——16. *Remarks on the Cattle of Sologne.* *By* Abbé Teſſier.——17. *Experiments on ſenſibility, reſpiration, and the anatomy of the Uterus, in the females of Quadrupeds.* *By* M. Vicq D'Azyr. In the diſorder that prevailed among the horned cattle in different provinces of France, it having been judged neceſſary to ſacrifice a great number, our author took this opportunity of making a variety of experiments on ſenſibility in general; on the ſenſibility of membranes and tendons; on the irritability of the iris; on the intercoſtal muſcles; on the bronchi of the fœtus; on the periſtaltic motion of the inteſtines; and on the uterus of cows.——18. *Memoir on the Regeneration of Bones. By* M. Troja. In a work entitled *de Novorum Oſſium regeneratione experimenta*, our author gave an account of a mode of producing the regeneration of long bones by deſtroying their marrow. As the greater number of his experiments on this ſubject were made upon pigeons, and only a few upon dogs, at the requeſt of M. Duhamel he made a greater number upon quadrupeds. Theſe experiments are related in the paper before us, and prove, that by deſtroying the marrow of a cylindrical bone, a new bony cylinder will be produced, either entire or partial, according as the whole or only a part of the marrow is removed, and which will correſpond with that part of the marrow which is deſtroyed.——19. *Reflections on the beſt manner of withdrawing M. Mejean's probe through the noſtrils in the operation for a fiſtula lachrymalis. By* M. Vicq D'Azyr. In 1712 M. Anel invented a method of curing the fiſtula lachrymalis, by introducing very ſlender probes through the puncta lachrymalia into the ſac, and injecting a fluid through the latter into the noſe by means of a ſmall ſyringe. Since his time, M. Mejean, of Montpellier, has improved this practice, by using probes which are perforated like a needle, and are long enough to extend from the upper *punctum* to the nose. By means of these, he introduces a bougie into the lachrymal duct. The difficulty in this operation lies in withdrawing the probe from the nose. With a view to obviate this inconvenience, the ingenious author of the paper before us recommends an instrument made of silver, rounded at its extremity, and having a groove extending through its whole length like a director, in which there are several holes large enough to admit the end of the probe. This instrument is to be introduced into the nose in search of the probe, which is to be moved gently upwards and downwards till its extremity gets into one of the holes, and then it is to be withdrawn.——20. *An account of a Method of improving the Preparation and Use of Emetic Tartar. By* M. de Lassone.——Our author prefers the mode of preparing this medicine recommended by Macquer in his chemical dictionary; but there is an objection, he says, to this as well as to every other, which is, that when dissolved in a very diluted aqueous vehicle, part of the medicine is constantly precipitated, and adheres to the sides of the phial. As this is owing to its not being sufficiently soluble, he recomrecommends the mixing equal quantities of emetic tartar and pure ſal ammoniac, and after rubbing them together in a mortar, adding a ſmall quantity, three parts or leſs, of diſtilled water. By this ſimple method, we are told, the two ſalts unite, and are completely diſſolved. A caſe of obſtinate double tertian is related, in which ſmall doſes of emetic tartar were given for ſeveral weeks as an alterative with a good effect. ——21. *An Eſſay on Acid Soaps, and on the advantages that may be derived from them in the practice of Phyſic. By* M. Macquer. The name of *ſoap* is now pretty generally applied to any combination of oil with a ſaline ſubſtance, by which the oil is rendered ſoluble either in water or ſpirit of wine. If this definition is adopted, the number of ſoaps that may be made will be almoſt infinite. Hitherto, however, hardly any other than the alkaline ſoaps have been much known. It is only ſince the Academy of Dijon announced this as a ſubject for a prize, that the attention of chemiſts has been directed to it. M. Achard's was one of the firſt and moſt intereſting publications on this matter. In uniting the acid of vitriol and oil, that writer recommends the acid to be concentrated, and the oil boiling hot, by which means the ſoap is black. To avoid this, this, our author advises the oil to be cold, and the acid diluted with a sufficient quantity of water, to prevent the mixture from becoming hot. By this method he thinks we may be able to procure a soap as white as the alkaline. The observations on its medicinal uses are merely speculative.——22. *An Essay on the Waters of La Preste in Roussillon. By* M. Bonifos. These waters, which are situated two leagues from Prali de Molo, and twelve from Perpignan, are said to be similar and no way inferior to those of Bareges and Bagneres. There are three springs there of different temperatures. In one, de Reaumur's thermometer pointed to 38½, in another to 36, and in the third to 25 degrees.——23. *Observations on the Analysis of Opium. By* M. Bucquet. This writer's experiments confirm the accounts of Neuman, Cartheuser, and Baumé with regard to the principles of opium. His method of procuring the extract is very simple. After grossly powdering the opium in a marble mortar, he pours cold water on it very gradually, and by gentle trituration promotes a solution. When the water is well tinged, he decants it and adds fresh, repeating this operation till the water comes away colourless, which it does when the opium has lost about half of its weight.

The

The liquor is then to be evaporated to the confiftence of an extract. The fubftance that remains in the mortar is a foft refinous matter, which preferves the pungent fmell of opium, and may be almoft wholly diffolved by fpirit of wine. ——24. *Analyfis of the Mineral Waters of Fontenelles, Broiffardiere, Reaumur, Boiffe, and Ramée, all in Lower Poitou. By* M. Gallot.——25. *Obfervations on Smutty Rye, by* Abbé Teffier. This is the *clavus fecalinus, fecalis mater* of Thalius, Ray, and others. Thefe fmutty grains abound more in fome years than others. They have been obferved in other plants as well as rye. Sologne is the part of France in which they are moft prevalent. There are two reafons affigned for this by our author; one is, that rye is more cultivated in that than in any other province, and the other, that the foil, which is in general wet, feems particularly calculated to produce them.——26. *An account of a particular order of Mufhrooms, which may be called* bulbous. *By* M. Paulet. All the mufhrooms of this genus, according to our author, are of an unwholefome nature. Sometimes, he obferves, their effects are confined to the *primæ viæ*, and they excite only colic and diarrhœa; but in general they produce more alarming and dangerous fymptoms, toms, which are preceded by languor and anxiety. He divides them into two species of *fungi speciosi* and *fungi muscarii*. A minute description, with engravings, is given of each species.——27. *Two Memoirs on Medical Electricity. By* M. Mauduyt. ——The volume closes with *a Dissertation on the circumstances in Exanthematous Fevers, in which a cool regimen is preferable to a hot one.* This judicious performance obtained the premium offered by the Society in 1776, and is written by M. Jaubert, physician at Aix. The author very properly considers the miliary eruption as an accidental symptom, the offspring of a hot regimen. He criticises a modern French writer, who asserts that a patient may have the small-pox without any eruption. Without this criterion, he asks, how can we say that the patient had the variolous fever? He observes that the virus of the small-pox attacks the *tela cellulosa*, while that of the measles particularly affects the membranes. The former, he adds, excites phlegmonic and the latter a sort of erysipelatous inflammation, which proves that each of these diseases has its peculiar modification dependent on its virus. Acidulated drink, he remarks, is less proper in measles than in the small-pox; and cold air, which is so necessary in the latter, may be dangerous gerous in the former diſeaſe. The ſecondary fever of the meaſles is inflammatory, while that of the ſmall-pox is of a putrid nature. The whole of the diſſertation is worthy of attention.

